# Pancreatic and snake venom presynaptically active phospholipases A_2_ inhibit nicotinic acetylcholine receptors

**DOI:** 10.1371/journal.pone.0186206

**Published:** 2017-10-12

**Authors:** Catherine A. Vulfius, Igor E. Kasheverov, Elena V. Kryukova, Ekaterina N. Spirova, Irina V. Shelukhina, Vladislav G. Starkov, Tatyana V. Andreeva, Grazyna Faure, Marios Zouridakis, Victor I. Tsetlin, Yuri N. Utkin

**Affiliations:** 1 Institute of Cell Biophysics, Russian Academy of Sciences, Pushchino, Moscow Region, Russia; 2 Shemyakin-Ovchinnikov Institute of Bioorganic Chemistry, Russian Academy of Sciences, Moscow, Russia; 3 Unité Récepteurs-Canaux, Institut Pasteur, Paris, France; 4 Hellenic Pasteur Institute, Athens, Greece; Weizmann Institute of Science, ISRAEL

## Abstract

Phospholipases A_2_ (PLA_2_s) are enzymes found throughout the animal kingdom. They hydrolyze phospholipids in the *sn*-2 position producing lysophospholipids and unsaturated fatty acids, agents that can damage membranes. PLA_2_s from snake venoms have numerous toxic effects, not all of which can be explained by phospholipid hydrolysis, and each enzyme has a specific effect. We have earlier demonstrated the capability of several snake venom PLA_2_s with different enzymatic, cytotoxic, anticoagulant and antiproliferative properties, to decrease acetylcholine-induced currents in *Lymnaea stagnalis* neurons, and to compete with α-bungarotoxin for binding to nicotinic acetylcholine receptors (nAChRs) and acetylcholine binding protein. Since nAChRs are implicated in postsynaptic and presynaptic activities, in this work we probe those PLA_2_s known to have strong presynaptic effects, namely β-bungarotoxin from *Bungarus multicinctus* and crotoxin from *Crotalus durissus terrificus*. We also wished to explore whether mammalian PLA_2_s interact with nAChRs, and have examined non-toxic PLA_2_ from porcine pancreas. It was found that porcine pancreatic PLA_2_ and presynaptic β-bungarotoxin blocked currents mediated by nAChRs in *Lymnaea* neurons with IC_50_s of 2.5 and 4.8 μM, respectively. Crotoxin competed with radioactive α-bungarotoxin for binding to *Torpedo* and human α7 nAChRs and to the acetylcholine binding protein. Pancreatic PLA_2_ interacted similarly with these targets; moreover, it inhibited radioactive α-bungarotoxin binding to the water-soluble extracellular domain of human α9 nAChR, and blocked acetylcholine induced currents in human α9α10 nAChRs heterologously expressed in *Xenopus* oocytes. These and our earlier results show that all snake PLA_2_s, including presynaptically active crotoxin and β-bungarotoxin, as well as mammalian pancreatic PLA_2_, interact with nAChRs. The data obtained suggest that this interaction may be a general property of all PLA_2_s, which should be proved by further experiments.

## Introduction

Phospholipases A_2_ (PLA_2_s, phosphatidylcholine 2-acylhydrolase, EC 3.1.1.4) hydrolyze predominantly phospholipids with polyunsaturated fatty acid residue in the *sn*-2 position; they are therefore essential participants in lipid digestion. In addition, they are involved in a range of other cell processes including inflammation, cell proliferation and signal transduction, largely because of their phospholipolytic activity [1]. The PLA_2_ superfamily includes 15 groups comprising four main types including the secreted, cytosolic, calcium-independent PLA_2_s, and platelet activating factor acetyl hydrolase/oxidized lipid lipoprotein-associated PLA_2_ [2]. Group I PLA_2_s are present in Elapidae snake venoms as group IA and in pancreatic juices of animals as group IB. The most obvious difference between them is the absence (group IA) or the presence (group IB) of an extra amino acid fragment, known as a pancreatic loop, next to the catalytic active site. Group II PLA_2_s are present in Viperidae snake venoms and in the synovial fluids of animals. These PLA_2_s, which are secreted by venomous glands of snakes, bees and other venomous animals, manifest various toxic actions. PLA_2_s from snake venoms have numerous toxic effects, not all of which can be explained by phospholipid hydrolysis, and each individual enzyme may have a specific effect. Some PLA_2_s are characterized by potent anticoagulant activity, for example PA11 from *Pseudechis australis* venom [3]; others manifest strong myotoxic properties, such as Lemnitoxin from *Micrurus lemniscatus* venom [4]. Among numerous PLA_2_ effects, neurotoxic action is one of the most important. Neurotoxicity is due to the block of neuromuscular transmission and proceeds in several steps: an initial weak inhibition of acetylcholine (ACh) release; a more prolonged facilitation of ACh secretion; and then a progressive decline of transmission leading to irreversible arrest [5–7].

There are several hypotheses of the mechanism of PLA_2_ neurotoxic action.

1. Phospholipolytic damage to the presynaptic membrane potentiates fusion of ready-to release synaptic vesicles in the active zone of neuroexocytosis, and inhibits vesicle retrieval [6]; consequently the ACh store is depleted.
2. Interaction of PLA_2_s with specific proteins: binding to these receptors facilitates a local enzyme-dependent or independent action [8]; the discovery of proteins that bind PLA_2_ with high affinity in different tissues supports this hypothesis [9–12].
3. Interaction of PLA_2_s with intracellular Ca^2+^ binding proteins after endocytosis or penetration through damaged membranes causing an increase in intracellular Ca^2+^ concentration, both leading to mitochondrial uncoupling [7, 13].

We have earlier reported antagonistic action of eight PLA_2_s from the venoms of snakes of Viperidae and Elapidae families (PLA_2_ groups IIA and IA, respectively) on nicotinic acetylcholine receptors (nAChRs) of different types [14, 15]. These enzymes, which differ in their enzymatic activities, competed with [^125^I]α-bungarotoxin (α-Bgt) for binding to the muscle-type nAChRs of *Torpedo californica* electric organ, to human α7 nAChRs expressed in GH_4_C_1_ cell line, and to ACh-binding protein (AChBP) from *Lymnaea stagnalis*. When tested on isolated neurons of *L*. *stagnalis* which contain α7 similar nAChRs [16, 17], PLA_2_s suppressed ACh- or cytisine-evoked currents under conditions that exclude hydrolysis of membrane phospholipids. These results indicate that binding of PLA_2_s to nAChRs affects their function.

To ascertain whether all types of PLA_2_s are able to interact with nAChRs, we have studied the action of three other phospholipases i.e. presynaptically active β-bungarotoxin (β-Bgt) from *Bungarus multicinctus*, crotoxin (Cro) from *Crotalus durissus terrificus* snake venom, and non-toxic mammalian PLA_2_ from porcine pancreas (PP PLA_2_, group IIB)—in binding assay and on *Lymnaea* neurons. β-Bgt is a heterodimeric protein in which a group IA PLA_2_ and a Kunitz type serine protease inhibitor are connected by a disulfide bond [18]. Cro is also a heterodimeric protein, and consists of a weakly toxic basic group IIA PLA_2_ and crotapotin, a non-enzymatic, non-toxic acidic component [19]. Mammalian porcine pancreatic PP PLA_2_ has been shown previously to induce presynaptic block of neuromuscular transmission in a mouse hemi-diaphragm preparation although it was much weaker than snake venom PLA_2_s [20]. β-Bgt and Cro have been previously shown to act presynaptically in a mammalian neuromuscular junction preparation [5, 21, 22].

We found that all PLA_2_s tested in this work interacted with nAChRs, with IC_50_ values ranging from hundreds of nM to tens of μM. The data from Cro revealed the presence of two sites both in muscle-type and α7 nAChRs with affinities differing by 1–3 orders of magnitude. Thus, we conclude that presynatically active PLA_2_s interact with muscle type nAChRs located postsynaptically. Moreover, it is not only snake venom PLA_2_s that are capable of binding to nAChRs, but mammalian pancreatic PLA_2_ also has this ability.

## Materials and methods

PLA_2_ from porcine pancreas (PP PLA_2_), Trizma-HCl, EGTA, HEPES, β-lactoglobulin, Pronase E, acetylcholine iodide, cytisine, choline chloride, and all chloride salts were purchased from Sigma (USA). RNAse was from P-L Biochemicals, Inc. (USA), soybean trypsin inhibitor from Boehringer Mannheim GmbH (Germany), cytochrome C from Ferak Berlin. Crotoxin from *Crotalus durissus terrificus* venom was purified as previously described [23, 24]. β-Bgt was isolated from *Bungarus multicinctus* venom by procedure described in [25]. Mono-iodinated (3-[^125^I]iodotyrosyl^54^)-α-Bgt (~2000 Ci/mmol) was from GE Healthcare. nAChR-enriched membranes from the electric organs of *T*. *californica* ray were kindly provided by Prof. F. Hucho (Free University of Berlin, Germany), GH_4_C_1_ cells transfected with human α7 nAChR were a gift from Eli-Lilly (USA). The expressed acetylcholine binding protein (AChBP) from *L*. *stagnalis* was kindly provided by Prof. T. Sixma (Netherlands Cancer Institute, Amsterdam, the Netherlands); the extracellular domain (ECD) of the human neuronal α9 nAChR was expressed, enzymatically deglycosylated and purified as described [26]. Plasmid pT7TS constructs of human nAChR α9 and α10 subunits were kindly provided by Prof. D.J.Adams (University of Wollongong, Wollongong, Australia).

### Electrophysiological measurements

#### Identified *L*. *stagnalis* giant neurons

Pond snails *L*. *stagnalis* (3–4 cm long) were collected from lakes near the Oka River (Pushchino, Moscow region) and kept in tap water at 4–6°C until use. *L*. *stagnalis* has the conservation status “Least Concerned” and does not require a special permission for use. The experiments were carried out on identified giant neurons (LP1,2,3, RPV2,3; according to the map of *L*. *stagnalis* ganglia [27]) isolated from the left and right parietal ganglia as described [17]. Neurons were internally perfused with internal solution (in mM: CsCl 95, CaCl_2_ 0.3, EGTA 2, HEPES 10, pH 7.2) and voltage-clamped at –60 mV [28]. Constant flow of the external solution (in mM: NaCl 92, KCl 1.6, BaCl_2_ 2, MgCl_2_ 1.5, Trizma-HCl 4, pH 7.6; Ba^2+^ was used instead of Ca^2+^ to avoid phospholipolytic action of the PLA_2_s on the cell membrane) was maintained, except the time of application of an agonist or neuron incubation with PLA_2_s. In the experiments with proteins lacking phospholipolytic activity, the CaCl_2_-containing extracellular solution was used. Acetylcholine (ACh), cytisine (Cyt) or choline were applied on the whole cell surface using 4 s pulses with intervals not less than 6 min. Agonist-induced currents were monitored and digitized with a patch-clamp amplifier A-M Systems (USA), the data acquisition was performed using Digidata1200 B interface and pClamp6 software (Axon Instruments Inc., USA). Aliquots of solutions of PLA_2_s in water were kept in the refrigerator and diluted using extracellular solution to the desired concentration immediately before use.

The effects of PLA_2_s were determined by measuring the changes in peak current amplitude induced by the agonist after 5-min incubation with PLA_2_ compared to the control responses before treatment and after prolonged washing. IC_50_ values were calculated using Sigma plot 11.0 software using the Hill plot analysis.

#### *Xenopus* oocytes

Plasmid pT7TS constructs of human nAChR α9 and α10 subunits were linearized with *Xba*I restriction enzymes (NEB, USA). Linearized plasmid constructs were subjected to *in vitro* cRNA transcription using T7 mMessagemMachine® transcription kit (AMBION, USA).

Mature *Xenopus laevis* female frogs used in this study were obtained commercially (NASCO, Fort Atkinson, WI, USA) and housed in a facility with 12:12 hours light:dark cycles, 18–20°C ambient temperature. Animals were fed twice a week and maintained according to supplier recommendations (https://www.enasco.com/page/xen_care). All the appropriate actions were taken to minimize discomfort to frogs. The World Health Organization’s International Guiding Principles for Biomedical Research Involving Animals were followed during experiments on animals. Oocytes were prepared from mature female frogs by following the standard procedure described elsewhere [29]. Stage V-VI oocytes were defolliculated with 2 mg/mL collagenase Type I (Life Technologies, USA) at room temperature (21−24°C) for 2 h in ND96 solution composed of (in mM) 96 NaCl, 2 KCl, 1 CaCl_2_, 1 MgCl_2_ and 5 HEPES at pH 7.4. Oocytes were injected with 9.2 ng of human nAChR α9 and α10 cRNA (in a ratio 1:1) and incubated at 18°C in Barth’s solution composed of (in mM) 88 NaCl, 1.1 KCl, 2.4 NaHCO_3_, 0.3 Ca(NO_3_)_2_, 0.4 CaCl_2_, 0.8 MgSO_4_ and 15 HEPES-NaOH at pH 7.6, supplemented with 40 μg/mL gentamicin and 100 μg/mL ampicillin for 4 days before electrophysiological recordings.

Two-electrode voltage clamp recordings at a holding potential of -60 mV were made using turbo TEC-03X amplifier (Npi electronic, Germany) and WinWCP recording software (University of Strathclyde, UK). Oocytes were briefly washed with Ba^2+^ Ringer’s solution (in mM: 115 NaCl, 2.5 KCl, 1.8 BaCl_2_, 10 HEPES at pH 7.2) followed by 3 applications of 25 μM ACh. Washout with Ba^2+^ Ringer’s solution was done for 5 min between ACh applications. Oocytes were incubated with PP PLA_2_ for 5 min followed by its co-application with ACh. Peak current amplitudes of ACh-induced responses were measured before and after preincubation of oocytes with PP PLA_2_. The ratio between these two measurements was used to assess the activity of PLA_2_ on human α9α10 nAChR.

### Receptor binding studies

#### Radioligand analysis

For competition binding assays, suspensions of nAChR-rich membranes from *T*. *californica* ray electric organ (1.25 nM α-Bgt binding sites) in 20 mM Tris-HCl buffer, pH 8.0, containing 1 mg/ml bovine serum albumin (BSA) (binding buffer), human α7 nAChR transfected GH_4_C_1_ cells (0.4 nM α-Bgt binding sites) in binding buffer, or a solution of heterologously expressed AChBP from *L*. *stagnalis* (2.4 nM in binding buffer) were incubated for 3 h with various amounts of the PLA_2_s, followed by an additional 5 min incubation with 0.4 nM [^125^I]α-Bgt. Nonspecific binding was determined by preliminary incubation of the preparations with 20 μM α-cobratoxin. The membrane and cell suspensions were applied to glass GF/C filters (Whatman, Little Chalfont, UK) presoaked in 0.25% polyethylenimine, and the unbound radioactivity was removed from the filter by washing (3 × 3 ml) with 20 mM Tris-HCl buffer, pH 8.0, containing 0.1 mg/ml BSA (washing buffer). The AChBP solutions were applied to two layers of DE-81 filters presoaked in PBS-T buffer, and washed (3 × 3 ml) with washing buffer. The bound radioactivity was determined using a Wizard 1470 Automatic Gamma Counter (Perkin Elmer). The binding results were analyzed using ORIGIN 7.5 (OriginLab Corporation, Northampton, MA, USA) fitting to a one-site or two-site dose-response competition curve.

For competition binding assays on the extracellular domain (ECD) of human α9 nAChR, PP PLA_2_ in the concentration range 0.3–30 μM was incubated for 2 h at room temperature with the ECD (final concentrations of 30 μg/ml) in 50 μL of a 20 mM Tris–HCl buffer, pH 8.0, containing 1 mg/ml of the bovine serum albumin (binding buffer). Then [^125^I]α-Bgt was added to the reaction mixtures to a final concentration of 0.2 nM. Simultaneously 15 μL Ni-NTA-agarose (QIAGEN) pre-washed in reaction buffer was added. After 6 min, the reaction was stopped by a rapid filtration on GF/C filters (Whatman) pre-soaked in 0.25% polyethylenimine and the unbound radioactivity was removed from the filters by washes (3×4 ml) with the 20 mM Tris–HCl buffer. Nonspecific binding was determined by preliminary incubation of the ECD with 10 μM α-cobratoxin. The bound radioactivity was determined using Wizard 1470 Automatic Gamma Counter (Perkin Elmer). The data were analyzed using ORIGIN 7.5 as a one-site dose-response curve.

#### Surface plasmon resonance (SPR) experiments

SPR experiments were performed at 20°C, using a Biacore^®^ 2000 system (GE Healthcare, Biacore AB). AChBP was covalently immobilized to a CM5 sensor chip at acidic pH. For binding experiments, the running and dilution buffer was composed of 20 mM Tris (pH 7.4), 150 mM NaCl, and 0.005% Surfactant P20 (GE Healthcare, Biacore AB). The concentrations of Cro ranged from 5.5 to 46 μg/ml, and solutions were injected at a flow rate of 30 μl/min. Background signals were obtained by injection of samples to a blank-immobilized flow cell and these signals were subtracted from the sample signals. At the end of each run, a 10 s injection of 10 mM Gly/HCl pH 1.5 was performed to restore the complete binding capacity of the AСhBP coupled to the CM5 sensor chip. The kinetic constants, k_a_ (association rate constant), and k_d_ (dissociation rate constant), for the interaction between Cro and AChBP were calculated using Biacore BIAEVALUATION 3.1 software (Biacore AB). The curves were fitted according to the simple two-component model of interaction. The apparent dissociation constant (K_D_^app^) was obtained as the ratio of k_d_ and k_a_ (K_D_^app^ = k_d_/k_a_).

## Results

### Electrophysiological experiments

#### Suppression of acetylcholine- or cytisine-induced currents in *L*. *stagnalis* neurons

Under the experimental conditions used, ACh and cytisine elicited inward currents in identified *L*. *stagnalis* neurons LP1,2,3 and RPV2,3 due to an increase in chloride permeability [30]. Previously this conductance was shown to be mediated by two subtypes of nAChRs with low and high affinity for α-conotoxin ImI (ImI) and reversed relative affinities for ACh [15, 17]. A further distinction between two subtypes is in the kinetics of receptor desensitization in response to ACh. The nAChRs with a higher sensitivity to ImI and faster desensitization, in spite of possessing chloride ion conductance, are more similar to vertebrate α7 nAChRs; furthermore cytisine is a full agonist at this subtype whereas it is a weak partial agonist at other nAChR subtype. In most experiments, we used cytisine or choline instead of ACh because of their more selective actions on α7 nAChRs. To exclude a possible contribution of phospholipolytic activity, Ca^2+^ was replaced with Ba^2+^ in the extracellular solution.

**Fig 1:**
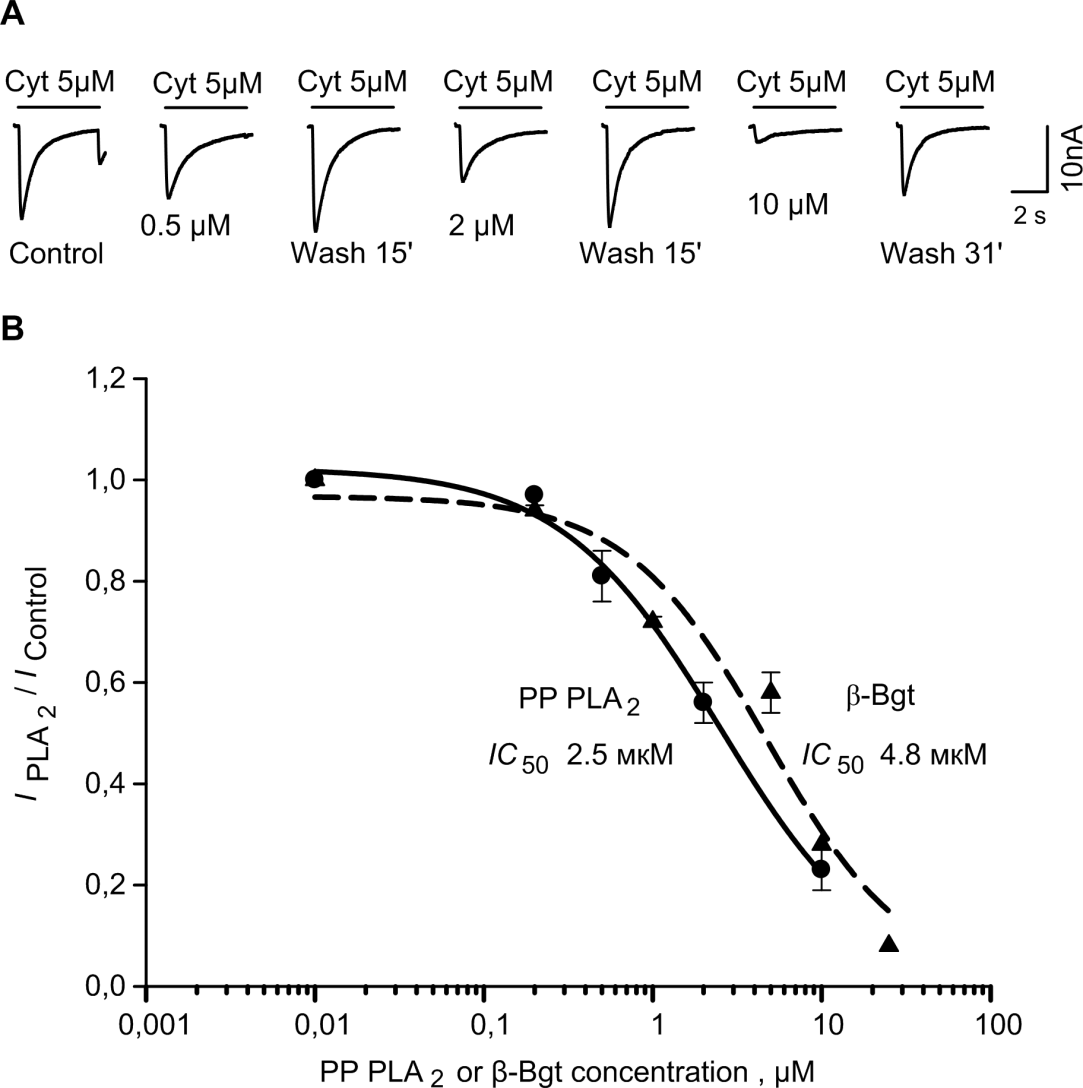
Inhibition of acetylcholine or cytisine-elicited current in *L*.*stagnalis* neurons by PP PLA_2_ and β-Bgt. (A) Representative recordings from a neuron in control, after a 5-min incubation with PP PLA_2_ at three different concentrations, and after PP PLA_2_ wash out. (B) Dependence of ACh- or cytisine-evoked current suppression on PP PLA_2_ (n = 7) and β-Bgt (n = 4) concentration.

It was found that 5 min treatment of a neuron with PP PLA_2_ or β-Bgt resulted in a decrease of ACh- or cytisine-induced currents (Fig 1A). Peak response suppression was dependent on PLA_2_ concentration (Fig 1B) and reversed slowly after PLA_2_ wash out. IC_50_ values for PP PLA_2_ and β-Bgt inhibition of cytisine-induced current were 2.5 ± 0.4 (n = 7) and 4.8 ± 1.6 (n = 4) μM, respectively.

**Fig 2:**
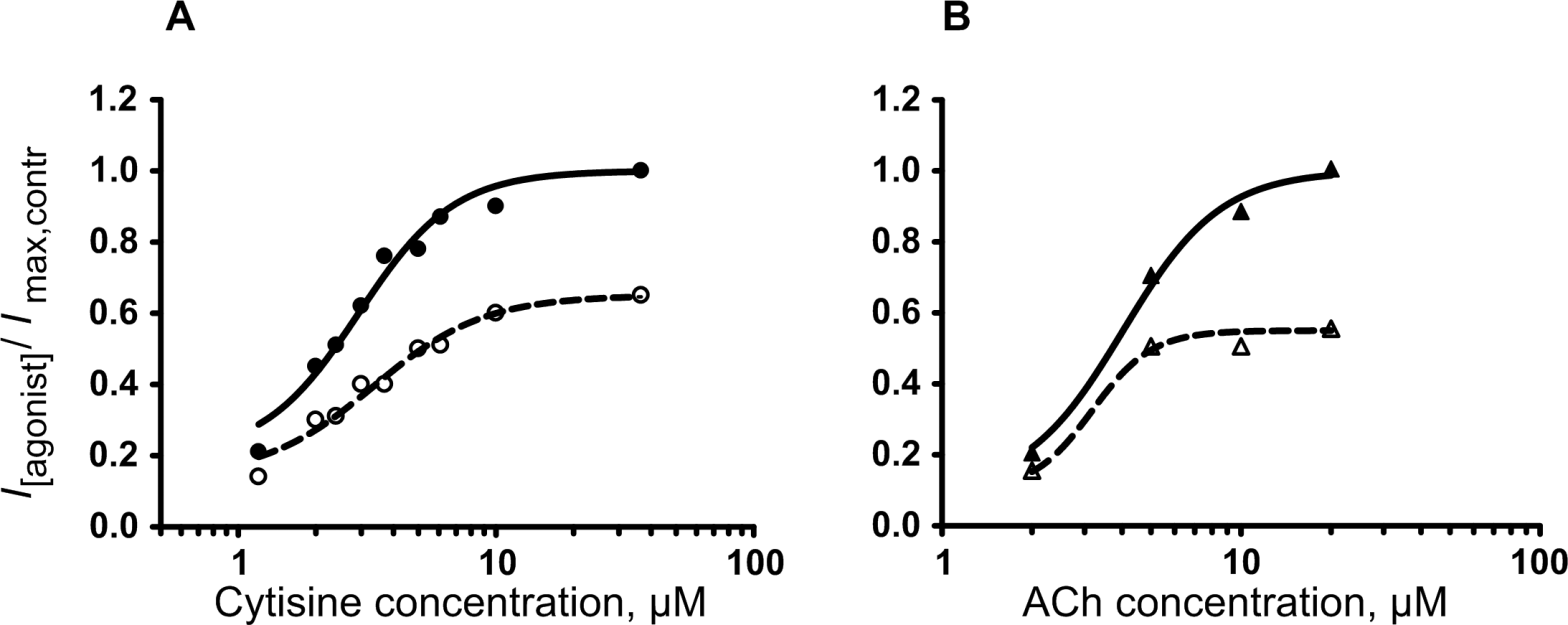
Determination of antagonism type for PP PLA_2_. Dependence of cytisine (A) or acetylcholine (B) induced currents on agonist concentration in control (closed circles and triangles) and after 5 min treatment with PP PLA_2_ at 3 μM (open symbols), (n = 5 and 1, respectively).

For PP PLA_2_ the type of antagonism was determined. For this purpose, a set of cytisine or ACh concentrations including saturating ones were applied to a neuron before and after treatment with PP PLA_2_ at 3 μM (a concentration slightly higher than its IC_50)_. As can be seen in Fig 2A and 2B, the curves of the current dependence on agonist concentration shifted rightward after incubation of the neurons with PP PLA_2_ solution. EC_50_ values for cytisine were 2.9 in control and 3.3 μM after PP PLA_2_ treatment (n = 4) and for ACh—4.0 and 3.2 μM (n = 1), respectively. However, the maximal responses to both cytisine and ACh were reduced by 40–50% (Fig 2). These data indicate non-competitive antagonism and support our previous results obtained with enzymatically inactive Vur-S49 from *Vipera ursinii renardi* venom [15].

We also explored the ability of the proteins unrelated to PLA_2_ to interact with nAChRs. Ribonuclease A (RNAse), β-lactoglobulin and soybean trypsin inhibitor were tested on *Lymnaea* neurons. We found that RNAse decreased the peak of the cytisine-induced current but the effect (IC_50_ > 50 μM, n = 3) was more than an order of magnitude weaker than that for PP PLA_2_ or β-Bgt. A decrease in the response to choline caused by β-lactoglobulin or soybean trypsin inhibitor at concentrations of 10 and 50 μM was not more than 8 and 12%, respectively (n = 7 and 7), and did not depend on the concentration of these proteins (data not shown).

#### Suppression of acetylcholine-induced current mediated by human α9α10 nAChR heterologously expressed in *Xenopus* oocytes

**Fig 3:**
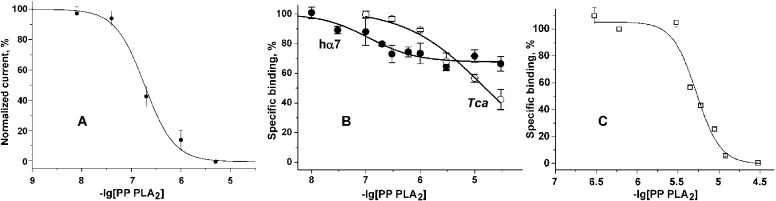
Inhibition experiments with PP PLA_2_. (A) Dose-response curve of PP PLA_2_ inhibitory action on the ACh-evoked (25 μM ACh) ionic currents mediated by human α9α10 nAChR heterologously expressed in *Xenopus* oocytes. (B) Inhibition by PP PLA_2_ of the initial rate of specific [^125^I]-α-Bgt binding to *T*. *californica* and human hα7 nAChRs expessed in GH_4_C_1_ cells. Only 30% of binding sites in hα7 nAChRs could be protected from [^125^I]-α-Bgt binding. (C) Inhibition by PP PLA_2_ of specific [^125^I]-α-Bgt binding to ECD of human α9 nAChR. IC_50_ 5.5 μM.

The activity of PP PLA_2_ was tested in electrophysiological experiments on human α9α10 nAChR heterologously expressed in *Xenopus* oocytes. It was found that a 5 min treatment of oocytes with PP PLA_2_ resulted in a decrease of the ACh-induced current (Fig 3A). Peak response suppression was dependent on PP PLA_2_ concentration (Fig 3A) and the IC_50_ value was 0.19 ± 0.03 μM (n = 3).

### Competition of PLA_2_s with [^125^I]α-bungarotoxin in binding assay

The capability of PLA_2_s to interact with nAChRs was studied using muscle-type nAChRs of *T*. *californica* electric organ and human neuronal α7 nAChRs (hα7 nAChRs) heterologously expressed in cells of GH_4_C_1_ line. ACh-binding protein (AChBP) from *L*. *stagnalis*, a structural analog of the extracellular ligand-binding domain of all nAChR subtypes, and the extracellular domain (ECD) of human α9 nAChR were also used for this purpose. The affinities of PLA_2_s for nAChRs, AChBP and ECD were evaluated using a radioligand competition binding assay with [^125^I]-labeled α-Bgt. The data obtained showed that all these PLA_2_s inhibited the initial rate of [^125^I]-labeled α-Bgt binding, although their potencies differed.

At *Torpedo* nAChR, PP PLA_2_ inhibited [^125^I]-α-Bgt binding with low efficiency (IC_50_ about 15 μM) with tend of complete inhibition at greater than 100 μM (Fig 3B). However, the inhibition of α-Bgt binding to hα7 nAChRs by PP PLA_2_ reached a plateau at approximately 70% of binding sites, the affinity to 30% of binding sites being fairly high (IC_50_ = 120 nM) (Fig 3B). Comparison of these results with the data on suppression of the cytisine-evoked current in *L*. *stagnalis* neurons indicates that both the affinities of PP PLA_2_ for hα7 and α7-similar nAChRs in *Lymnaea* neurons and the degree of inhibition of these receptors differed greatly. The interaction of PP PLA_2_ with ECD of human α9 nAChR showed one binding site with IC_50_ of 5.5 μM (Fig 3C).

**Fig 4:**
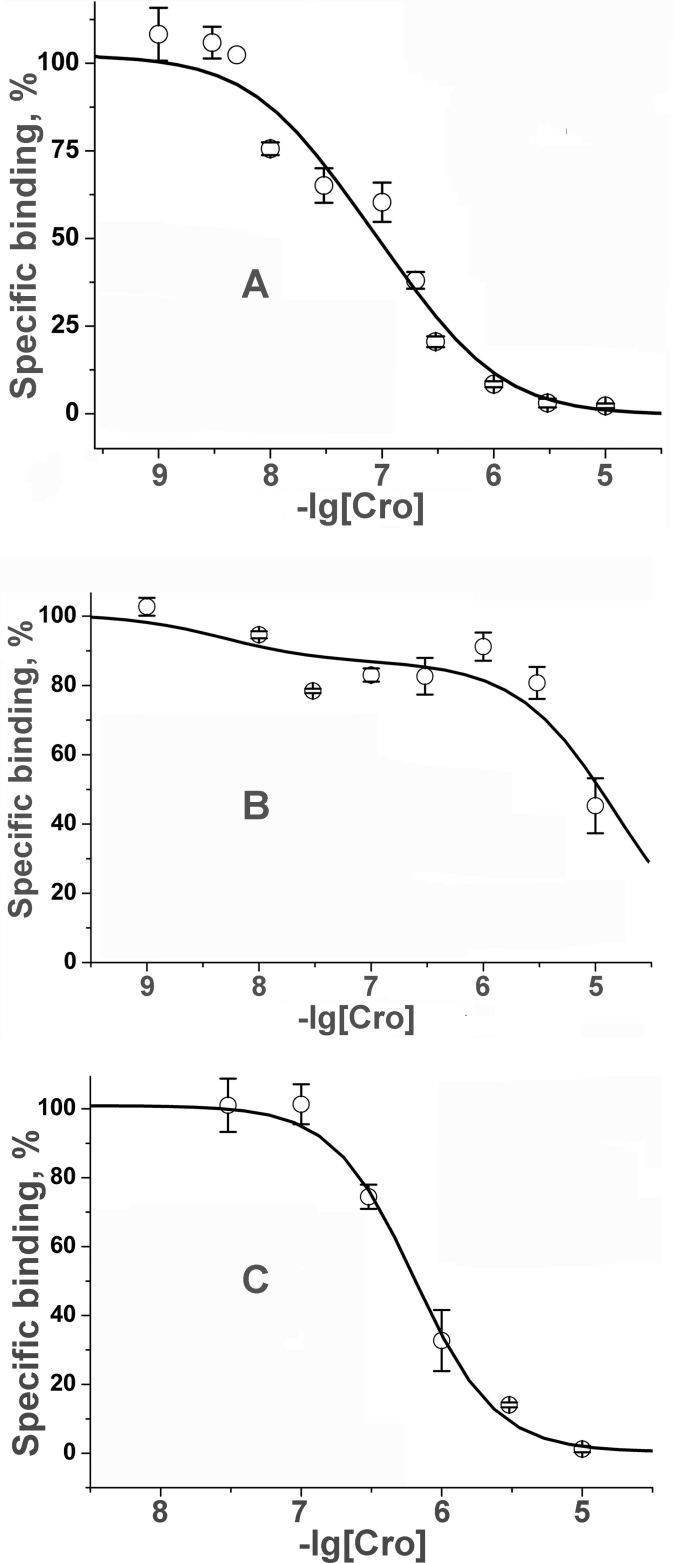
Interaction of Cro with nAChR of *T*. *californica* electric organ, human neuronal α7 nAChR and AChBP. (A) Inhibition of the initial rate of specific [^125^I]-α-Bgt binding to *T*.*californica* nAChRs by Cro. Points were fit to a 2-site model with affinity for Cro of 30 nM and 260 nM. (B) Inhibition of the initial rate of specific [^125^I]-α-Bgt binding to human α7 nAChRs by Cro. Two binding sites with affinity for Cro differing more than 3 orders of magnitude were revealed. (C) Inhibition of the initial rate of specific [^125^I]-α-Bgt binding to acetylcholine-binding protein from *L*. *staganlis* by Cro.

Cro completely inhibited [^125^I]-α-Bgt binding to all three targets (Fig 4). The experimental points for *Torpedo* and hα7 nAChRs were best approximated by a two-site model, with the difference in affinities between the two sites being about an order of magnitude in *Torpedo* nAChRs (30 and 260 nM) and more than 3 orders of magnitude in hα7 nAChRs (4.9 nM and 15 μM). For both the high affinity binding sites accounted for 20–30% of all binding sites. The interaction of Cro with AChBP showed one binding site with an IC_50_ of 640 nM (Fig 4C).

**Fig 5:**
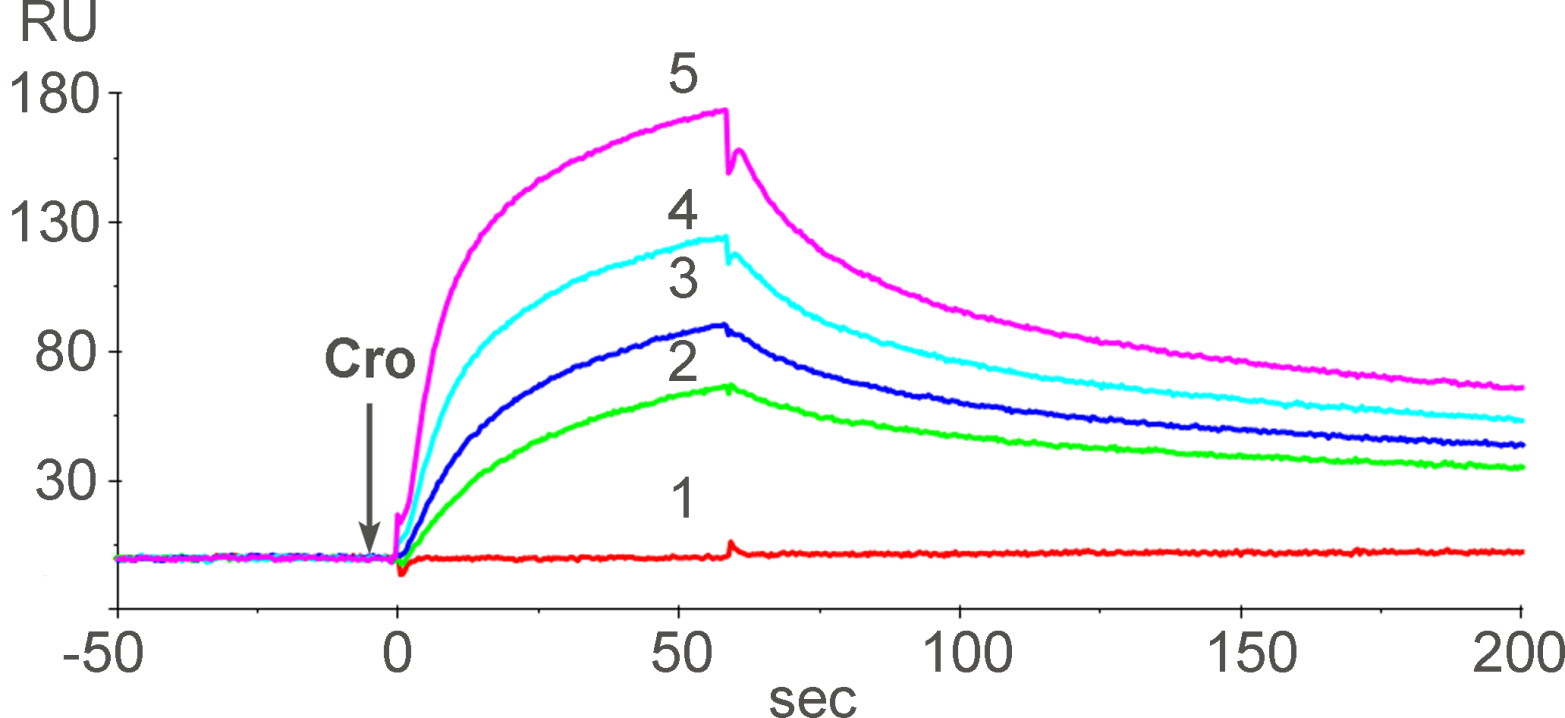
SPR recordings of Cro interaction with AChBP from *L*. *stanalis*. An arrow indicates injection of the analyte. Line 1 corresponds to injection of buffer solution. Curve 2–5 correspond to injections of solutions with Cro at concentrations of 5.5, 11.5, 23 and 46 μg/ml, respectively.

To observe the direct binding of Cro to AChBP, SPR measurements were performed (Fig 5). SPR recordings demonstrated that Cro interacted with immobilized AChBP and formed a stable complex. Quantitative analysis allowed the determination of the apparent dissociation constant (K_D_^app^) for this interaction which was 120 nM.

**Fig 6:**
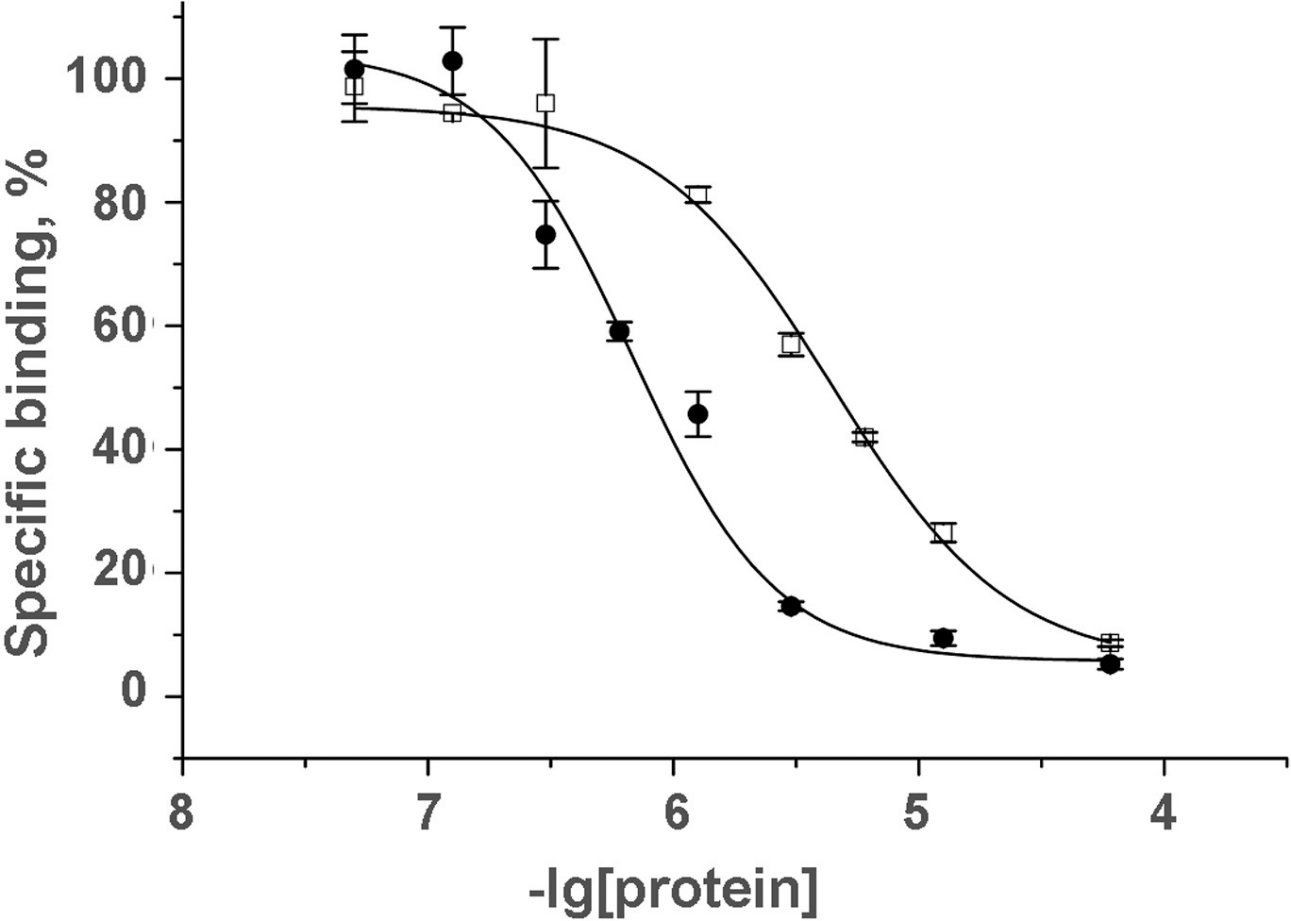
Interaction of non-venom proteins with *T*.*californica* nAChR. Inhibition of the initial rate of specific [^125^I]-α-Bgt binding to *T*.*californica* nAChR by RNAse (squares) and cytochrome C (circles).

Several other proteins were checked for inhibition of α-Bgt binding. The proteins chosen have molecular masses close either to monomer PLA_2_ (RNAse, cytochrome C) or to heterodimer (soybean typsin inhibitor). Soybean trypsin inhibitor and β-lactoglobulin were inactive at concentration up to 60 μM. These data coincide with the results on *Lymnaea* neurons. RNAse inhibited α-Bgt binding to *Torpedo* nAChRs fairly well (IC_50_ 5 μM) and cytochrome C was slightly more potent (IC_50_ 1.12 μM) (Fig 6). However, these proteins even at high concentrations had only marginal effects on function of nAChRs in *Lymnaea* neurons.

## Discussion

Snake venom PLA_2_s are multi-functional proteins evolved to affect multiple biological targets in prey organisms. They possess different toxic activities including presynaptic neurotoxicity, myotoxicity, cardiotoxicity, anticoagulant, and haemolytic activity. Usually each individual enzyme manifests its own specific effect, although other weaker activities can also be observed. For example, the presynaptically acting neurotoxin Cro has analgesic actions, and also immunomodulatory and anti-inflammatory effects [19], with no direct correlation between these activities and the catalytic activity of Cro. The PLA_2_ effects could be manifested through direct binding to membrane-bound receptors, and several binding proteins and glycoproteins, the so-called M- and N-receptors, which are tissue specific and bind certain PLA_2_s, have been discovered [9–12]. These receptors were shown to have high affinity binding sites for PLA_2_s with IC_50_ ranging from several pM to about 100 nM, i.e. significantly higher than the affinity of PLA_2_s for phospholipids [8]). One receptor protein isolated from porcine cerebral cortex bound not only neurotoxic PLA_2_s from snake venoms but also non-toxic PP PLA_2_ with similar affinities [12]. High affinity binding of mouse non-toxic IIA and IB PLA_2_s to M-type receptors in mouse colon has also been reported [31]. These data indicate that PLA_2_s possess the capacity to interact with receptor proteins.

In our previous paper, we reported on the ability of PLA_2_s from two families of snakes to antagonize an ACh-elicited current in *L*. *stagnalis* neurons containing α7-like receptors, and to compete with α-Bgt binding to recombinant human α7 nAChRs, *T*.*californica* nAChRs and AChBP from *L*. *stagnalis* [15]. Using Ca^2+^-free solution and experiments with a natural non-enzymatic analog of PLA_2_ from *V*. *ursinii renardi* venom (Vur-S49) allowed us to explore the type of interaction with nAChRs of different types. Although PLA_2_s completely inhibited α-Bgt binding to nAChRs and AChBP, characteristic changes of current-agonist concentration curves after neuron treatment with Vur-S49 indicated a non-competitive interaction.

In this paper, we present additional evidence for interaction with nAChRs of two dimeric presynaptic PLA_2_ toxins, i.e. Cro from *C*. *durissus terrificus* and β-Bgt from *B*. *multicinctus*, as well as non-toxic mammalian PLA_2_ from porcine pancreas. PP PLA_2_ and β-Bgt suppressed current responses of neurons to agonists with IC_50_ values of 2.5 and 4.8 μM, respectively (Fig 1). These values are in the same range as those obtained earlier for other PLA_2_s (0.4–10 μM [15]) which are monomeric enzymes. In heterodimeric β-Bgt the PLA_2_ subunit is connected to a Kunitz type inhibitor subunit by a disulfide bond, but this does not interfere with β-Bgt binding to nAChR. PP PLA_2_ suppressed the current responses of heterologously expressed human α9α10 nAChR to ACh with an IC_50_ value of 0.19 μM (Fig 3A). This is the highest affinity observed in electrophysiological experiments on current suppression by PLA_2_s.

In the binding assay, Cro and PP PLA_2_ competed with α-Bgt for binding to nAChR and AChBP. Interestingly, PP PLA_2_ at α7 nAChR competed with α-Bgt for binding to only about 30% of the total binding sites. The inhibition reached a plateau at about 3 μM and remained at the same level up to 30 μM (Fig 3B). The affinity for this 30% of sites was fairly high, the IC_50_ value being 150 nM. It should be noted that PP PLA_2_ completely inhibited the acetylcholine-induced current in *Lymnaea* neurons in a noncompetitive manner, albeit with a higher IC_50_ value (2.5 μM). The nAChR α7 subtype contains five α-Bgt binding sites [32], but α-Bgt binding to only one site is enough to block ion currents [33]. The bound α-Bgt locks the agonist-binding site in an inactive conformation and the dominant mechanism of antagonism is non-competitive, originating from conformational arrest of the binding sites [33]. Given these data, we suggest that at α7 nAChR, PP PLA_2_ competes with α-Bgt and binds to only one (or possibly two) binding site(s). However, PP PLA_2_ completely inhibits receptor ionic conductance in *Lymnaea* neurons. Therefore, binding of PP PLA_2_ to one or two sites could be sufficient to block *Lymnaea* receptor similar to the inhibition of α7 nAChR by α-Bgt.

PP PLA_2_ inhibited α-Bgt binding to the ECD of the human α9 nAChR with an IC_50_ value of 5.5 μM (Fig 3C), which is higher than that (0.19 μM) observed for inhibition of the ACh-induced current in oocytes expressing human α9α10 nAChR. Thus, for α9 nAChR we have observed receptor inhibition both in the ECD binding experiments and in electrophysiological experiments on whole receptors. It should be noted that signaling via α9α10 nAChRs is involved in the expression of pain [34] and inhibition of this receptor prevents neuropathic pain [35]. Consistent with these data, type IIA PLA_2_ has been localized by immunohistochemistry to the spinal trigeminal and facial motor nuclei and dorsal- and ventral-horns of the spinal cord [36], implying an important role of CNS sPLA_2_ in nociceptive transmission. It has also been shown that the treatment of mice with bee venom PLA_2_ might prevent oxaliplatin-induced neuropathic pain [37]. Given our data shows an interaction of PP PLA_2_ with α9α10 nAChR, we suggest that the PLA_2_ interaction with this receptor may be involved in the pain transmission pathway.

Interaction of Cro with muscle-type nAChRs has been previously studied [38, 39]. The toxin or phospholipolytically active basic component blocked depolarization and the Na^+^ permeability increase induced by carbamylcholine in membrane preparations from electric organs of *Electrophorus electricus* and *T*. *marmorata*. Although Cro reduced the initial velocity of labeled α-toxin from *Naja nigricollis* binding to postsynaptic membranes by about 30%, the authors concluded that Cro did not interfere with binding of α-toxin to nAChRs. These data along with Cro evoked enhancement of affinity to agonist was considered as a sign of non-competitive interaction of Cro with nAChR, leading to stabilization of the desensitized state [38]. In support of this, Cro decreased depolarization of the guinea-pig end-plate and the frequency of miniature end-plate postsynaptic potentials [39].

In this work, we found that Cro could compete with α-Bgt for binding to nAChRs. It is interesting to note that two binding sites with different affinities for Cro were revealed in both *Torpedo* and hα7 nAChRs: IC_50_ were of 30 and 260 nM for the first and 4.9 nM and 15 μM for the second (Fig 4). In *Torpedo* nAChR the ratio of low and high affinity binding sites was 1:1, corresponding to the presence of two agonist/competitive antagonist binding sites. Non-equivalence in the affinity of two binding sites of *Torpedo* nAChR was earlier shown for d-tubocurarine and α-conotoxins [40, 41]. In hα7 nAChR the high affinity binding sites represented about 20% of total sites. This finding could be explained by the assumption that the high affinity Cro binding to one site out of five ones resulted in some changes in the receptor which were responsible for a decrease in affinity to Cro in the other four sites.

In experiments with water soluble AChBP, Cro binding to one binding site was observed (Figs 4C and 5). The binding to water soluble protein observed both in competition with radioactive α-Bgt and direct SPR experiment allowed complete exclusion of membrane effects in its interaction.

Inhibition of α-Bgt binding to *Torpedo* nAChRs found in this study is consistent with Cro inhibition of responses to carbamylcholine observed in membrane preparations from electric organs and in guinea-pig diaphragm, although competitive binding of Cro and α-Bgt was not reported [38]. Our finding of muscle type nAChR inhibition by Cro demonstrates the postsynaptic activity of this toxin, although the block of hα7 nAChR by Cro may contribute to its presynaptic activity as the participation of α7 nAChR in acetylcholine release in mouse motor synapses was suggested [42].

According to our electrophysiological data, antagonism of PLA_2_s (Fig 2 here and Fig 4B in [15]) of nAChRs was non-competitive. This fact seems contradictory to the ability of all PLA_2_s to inhibit α-Bgt binding (Figs 3 and 4 here and Fig 6 in [15]). The reason of the discrepancy might be structural differences between α7 similar *Lymnaea* nAChRs and *Torpedo* or human α7 receptors. For another explanation of the PLA_2_ competition with α-Bgt, one should consider the existing model of α-neurotoxin-nAChR interaction: it is accepted that the tip of the toxin central loop is inserted into the receptor at the interfaces between two subunits and the toxin molecule is placed almost equatorially to the extracellular domain of the nAChR. Recently, a possible participation of the membrane in which nAChR is embedded in neurotoxin-receptor interaction was suggested [43, 44]. It was found that a snake venom neurotoxin can bind membrane and this binding can be considered as a first interaction step facilitating the receptor recognition by the toxin. Thus, it can be suggested that any disturbance of toxin interaction with the membrane can constrain its binding to the receptor. At the extreme, this may result in inhibition of toxin binding to the receptor. Interestingly, in competition experiments the interaction of Cro with the water soluble AChBP (Fig 4C) was weaker than with the membrane-bound nAChRs which could be due to participation of membrane in toxin-receptor interaction. Finally, competition with α-Bgt might be explained by PLA_2_ binding to nAChR in close vicinity to the agonist/competitive antagonist binding site that leads to steric hindrance of α-Bgt binding.

Here we found that PLA_2_s interact with nAChRs with different affinities, with IC_50_ ranging from tens of nM to tens of μM. The fairly low affinities in the micromolar range raise the question about specificity of interaction, and thus four proteins lacking phospholipolytic activity—RNAse, β-lactoglobulin, soybean trypsin inhibitor, and cytochrome C—were tested for their ability to interact with nAChRs. Unexpectedly, RNAse and cytochrome C could compete with α-Bgt for binding to *Torpedo* nAChR with IC_50_s of 1.12 and 5 μM, respectively (Fig 6), while soybean trypsin inhibitor and β-lactoglobulin did not compete with α-Bgt at concentrations up to 60 μM. However, all these proteins were practically inactive in functional tests on *Lymnaea* neurons: RNAse only slightly suppressed nAChR-mediated current with an IC_50_ greater than 50 μM, while soybean trypsin inhibitor and β-lactoglobulin were ineffective at this concentration. It is well documented that both RNAse [45] and cytochrome C [46] interact with cellular membranes, and in the view of the above consideration they might interfere with the α-Bgt binding to the membrane-associated nAChR. Indeed, such inhibition was observed in competition experiments with radioactive α-Bgt (Fig 6). However, the addition of RNAse had only marginal effect on the current elicited by agonist in *Lymnaea* neurons. The competition of the PLA_2_s with α-Bgt for binding to membranes could also explain the higher enzyme affinities observed in radioligand experiments as compared to electrophysiological data obtained in this and previous [15] work. PLA_2_s studied here not only competed with radioactive α-Bgt for binding to nAChRs but also blocked acetylcholine elicited ion currents.

In summary, we have revealed the ability of two toxic heterodimeric snake PLA_2_s and non-toxic PP PLA_2_ to interact with different types of nAChRs. These data indicate that the interaction with nAChR may be a general property of all PLA_2_s and defines a novel activity that can be attributed to these proteins.

## References

[1] Murakami M, Lambeau G. Emerging roles of secreted phospholipase A(2) enzymes: an update. Biochimie. 2013;95: 43–50. doi: 10.1016/j.biochi.2012.09.007 23022039 10.1016/j.biochi.2012.09.007

[2] Dennis EA, Cao J, Hsu YH, Magrioti V, Kokotos G. Phospholipase A2 enzymes: physical structure, biological function, disease implication, chemical inhibition, and therapeutic intervention. Chem Rev. 2011;111: 6130–6185. doi: 10.1021/cr200085w 21910409 10.1021/cr200085w PMC3196595

[3] Du QS, Trabi M, Richards RS, Mirtschin P, Madaras F, Nouwens A, et al Characterization and structural analysis of a potent anticoagulant phospholipase A2 from Pseudechis australis snake venom. Toxicon. 2016;111: 37–49. doi: 10.1016/j.toxicon.2015.12.017 26747471 10.1016/j.toxicon.2015.12.017

[4] Casais-E-Silva LL, Teixeira CF, Lebrun I, Lomonte B, Alape-Girón A, Gutiérrez JM. Lemnitoxin, the major component of Micrurus lemniscatus coral snake venom, is a myotoxic and pro-inflammatory phospholipase A2. Toxicol Lett. 2016;257: 60–71. doi: 10.1016/j.toxlet.2016.06.005 27282409 10.1016/j.toxlet.2016.06.005

[5] Rowan EG. What does β-bungarotoxin do at the neuromuscular junction? Toxicon. 2001;39: 107–118. 10936627 10.1016/s0041-0101(00)00159-8

[6] Montecucco C, Rossetto O, Caccin P, Rigoni M, Carli L, Morbiato L, et al Different mechanisms of inhibition of nerve terminals by botulinum toxin and snake presynaptic neurotoxins. Toxicon. 2009;54: 561–564. doi: 10.1016/j.toxicon.2008.12.012 19111566 10.1016/j.toxicon.2008.12.012

[7] Križaj I. Ammodytoxin: A window into understanding presynaptic toxicity of secreted Phospholipases A2 and more. Toxicon. 2011;58: 219–229. doi: 10.1016/j.toxicon.2011.06.009 21726572 10.1016/j.toxicon.2011.06.009

[8] Kini RM. Excitement ahead: structure, function and mechanism of snake venom phospholipase A2 enzymes. Toxicon. 2003;42: 827–840. doi: 10.1016/j.toxicon.2003.11.002 15019485 10.1016/j.toxicon.2003.11.002

[9] Lambeau G, Barhainin H, Schweitz H, Qar J, Lazdunski M. Identification and properties of very high affinity brain membrane-binding sites for a neurotoxic phospholipase from the taipan venom. J Biol Chem. 1989;264: 11503–11510. 2544597

[10] Lambeau G, Scmid-Alliana A, Lazdunsli M, Barhainin H. Identification and purification of a very high affinity binding protein for toxic phospholipases A2 in skeletal muscle. J Biol Chem. 1990;265: 9526–9532. 2160984

[11] Križaj I, Faure G, Gubenšek F, Bon C. Neurotoxic phospholipases A2 ammodytoxin and crotoxin bind to distinct high-affinity protein acceptors in *Torpedo marmorata* electric organ. Biochemistry. 1997;36: 2779–2787. doi: 10.1021/bi9612374 9062105 10.1021/bi9612374

[12] Čopič A, Vučemilo N, Gubenšek F, Križaj I. Identification and purification of a novel receptor for secretory phospholipase A2 in porcine cerebral cortex. J Biol Chem. 1999;274: 6315–26320. 10473587 10.1074/jbc.274.37.26315

[13] Rigoni M, Pizzo P, Schiavo G, Weston AE, Zatti G, Caccin P, et al Calcium influx and mitochondrial alterations at synapses exposed to snake neurotoxins or their phospholipids hydrolysis products. J Biol Chem. 2007;282: 11238–11245. doi: 10.1074/jbc.M610176200 17311918 10.1074/jbc.M610176200

[14] Vulfius CA, Gorbacheva EV, Starkov VG, Osipov AV, Kasheverov IE, et al An unusual phospholipase A_2_ from puff adder Bitis arietans venom–a novel blocker of nicotinic acetylcholine receptors. Toxicon. 2011;57: 787–793. doi: 10.1016/j.toxicon.2011.02.013 21333664 10.1016/j.toxicon.2011.02.013

[15] Vulfius CA, Kasheverov IE, Starkov VG, Osipov AV, Andreeva TV, Filkin SYu, et al Inhibition of nicotinic acetylcholine receptors, a novel facet in the pleiotropic activities of snake venom phospholipases A_2_. PLoS One. 2014;9: e115428 doi: 10.1371/journal.pone.0115428 25522251 10.1371/journal.pone.0115428 PMC4270787

[16] Vulfius CA, Krasts IV, Utkin YuN, Tsetlin VI. Nicotinic receptors in Lymnaea stagnalis neurons are blocked by neurotoxins from cobra venoms. Neurosci Lett. 2001;309: 189–192. 11514073 10.1016/s0304-3940(01)02081-x

[17] Vulfius CA, Tumina OB, Kasheverov IE, Utkin YuN, Tsetlin VI. Diversity of nicotinic receptors mediating Cl^−^ current in Lymnaea neurons distinguished with specific agonists and antagonist. Neurosci Lett. 2005;373: 232–236. doi: 10.1016/j.neulet.2004.10.010 15619549 10.1016/j.neulet.2004.10.010

[18] Kwong PD, McDonald NQ, Sigler PB, Hendrickson WA. Structure of beta -bungarotoxin: potassium channel binding by Kunitz modules and targeted phospholipase action. Structure. 1995;3: 1109–1119. 8590005 10.1016/s0969-2126(01)00246-5

[19] Sampaio SC, Hyslop S, Fontes MR, Prado-Franceschi J, Zambelli VO, Magro AJ, et al Crotoxin: novel activities for a classic beta-neurotoxin. Toxicon. 2010;55: 1045–1060. doi: 10.1016/j.toxicon.2010.01.011 20109480 10.1016/j.toxicon.2010.01.011

[20] Rigoni M, Caccin P, Gschmeissner S, Koster G, Postle AD, Rossetto O, et al Equivalent effects of snake PLA2 neurotoxins and lysophospholipid–fatty acid mixtures. Science. 2005;310: 1678–1680. doi: 10.1126/science.1120640 16339444 10.1126/science.1120640

[21] Chang CC, Lee CY. Crotoxin, the neurotoxin of South American rattlesnake venom is a presynaptic toxin acting like beta-bungarotoxin. Naunyn-Schemiedebergs Arch Pharmacol. 1977;296: 159–168. 10.1007/BF00508469 834316

[22] Prasarnpun S, Walsh J, Harris JB. Beta-bungarotoxin-induced depletion of synaptic vesicles at the mammalian neuromuscular junction. Neuropharmacology. 2004;47: 304–314. doi: 10.1016/j.neuropharm.2004.04.012 15223309 10.1016/j.neuropharm.2004.04.012

[23] Faure G, Bon C. Crotoxin, a phospholipase A2 neurotoxin from the South American Rattlesnake, Crotalus durissus terrificus: purification of several isoforms and comparison of their molecular structure and of their biological activities. Biochemistry. 1988;27: 730–738. 3349062 10.1021/bi00402a036

[24] Faure G, Xu H, Saul F. Crystal structure of crotoxin reveals key residues involved in stability and toxicity of this potent heterodimeric beta-neurotoxin. J Mol Biol. 2011;412: 176–191. doi: 10.1016/j.jmb.2011.07.027 21787789 10.1016/j.jmb.2011.07.027

[25] Utkin YN, Gantsova EA, Andreeva TV, Starkov VG, Ziganshin RH, Anh HN, et al Venoms of kraits Bungarus multicinctus and Bungarus fasciatus contain anticoagulant proteins. Dokl Biochem Biophys. 2015;460: 53–58. doi: 10.1134/S1607672915010159 25772992 10.1134/S1607672915010159

[26] Zouridakis M, Giastas P, Zarkadas E, Chroni-Tzartou D, Bregestovski P, Tzartos SJ. Crystal structures of free and antagonist-bound states of human alpha9 nicotinic receptor extracellular domain. Nat Struct Mol Biol. 2014;21: 976–980. doi: 10.1038/nsmb.2900 25282151 10.1038/nsmb.2900

[27] Benjamin PR, Ings CT. Golgi-Cox studies on the central nervous system of a gastropod mollusk. Z Zellforsch. 1972;128: 564–582. 4112391 10.1007/BF00306989

[28] Kostyuk PG, Krishtal OA, Pidoplichko VI. Intracellular perfusion. J. Neurosci Methods. 1981;4: 201–210. 6272031 10.1016/0165-0270(81)90032-7

[29] Kudryavtsev DS, Shelukhina IV, Son LV, Ojomoko LO, Kryukova EV, Lyukmanova EN, et al Neurotoxins from snake venoms and α-conotoxin ImI inhibit functionally active ionotropic γ-aminobutyric acid (GABA) receptors. J Biol Chem. 2015;290: 22747–22758. doi: 10.1074/jbc.M115.648824 26221036 10.1074/jbc.M115.648824 PMC4566246

[30] Chemeris NK, Kazachenko VN, Kislov AN, Kurchikov AL. Inhibition of acetylcholine responses by intracellular calcium in Lymnaea stagnalis neurons. J Physiol (London). 1982;323: 1–19. 6284913 10.1113/jphysiol.1982.sp014058 PMC1250342

[31] Cupillard L, Mulherkar R, Gomez N, Kadam S, Valentin E, Lazdunski M, et al Both group IB and group IIA secreted phospholipases A2 are natural ligands of the mouse 180 kDa M-type receptor. J Biol Chem. 1999;274: 7043–7051. 10066760 10.1074/jbc.274.11.7043

[32] Simonson PD, Deberg HA, Ge P, Alexander JK, Jeyifous O, Green WN, et al Counting bungarotoxin binding sites of nicotinic acetylcholine receptors in mammalian cells with high signal/noise ratios. Biophys J. 2010;99: L81–L83. doi: 10.1016/j.bpj.2010.08.076 21081055 10.1016/j.bpj.2010.08.076 PMC2980733

[33] daCosta CJ, Free CR, Sine SM. Stoichiometry for α-bungarotoxin block of α7 acetylcholine receptors. Nat Commun. 2015;6: 8057 doi: 10.1038/ncomms9057 26282895 10.1038/ncomms9057 PMC4544739

[34] Del Bufalo A, Cesario A, Salinaro G, Fini M, Russo P. Alpha9 alpha10 nicotinic acetylcholine receptors as target for the treatment of chronic pain. Curr Pharm Des. 2014;20: 6042–6047. 24641230 10.2174/1381612820666140314150634

[35] Romero HK, Christensen SB, Di Cesare Mannelli L, Gajewiak J, Ramachandra R, Elmslie KS, et al Inhibition of α9α10 nicotinic acetylcholine receptors prevents chemotherapy-induced neuropathic pain. Proc Natl Acad Sci U S A. 2017;114: E1825–E1832. doi: 10.1073/pnas.1621433114 28223528 10.1073/pnas.1621433114 PMC5347537

[36] Ma MT, Nevalainen TJ, Yeo JF, Ong WY. Expression profile of multiple secretory phospholipase A(2) isoforms in the rat CNS: enriched expression of sPLA(2)-IIA in brainstem and spinal cord. J Chem Neuroanat. 2010;39: 242–247. doi: 10.1016/j.jchemneu.2010.02.002 20153419 10.1016/j.jchemneu.2010.02.002

[37] Li D, Kim W, Shin D, Jung Y, Bae H, Kim SK. Preventive effects of bee venom derived phospholipase A₂ on oxaliplatin-induced neuropathic pain in mice. Toxins (Basel). 2016;8: E27. 26797636 10.3390/toxins8010027 PMC4728549

[38] Bon C, Changeux J-P, Jeng TW, Fraenkel-Conrat H. Postsynaptic effects of crotoxin and of its isolated subunits. Eur. J Biochem. 1979;99:471–481. 499210 10.1111/j.1432-1033.1979.tb13278.x

[39] Brazil OV, Fontana MD, Heluany NF. Nature of the postsynaptic action of crotoxin at guinea-pig diaphragm end-plates. J Nat Toxins. 2000;9: 33–42. 10701179

[40] Kreienkamp HJ, Utkin YN, Weise C, Machold J, Tsetlin VI, Hucho F. Investigation of ligand-binding sites of the acetylcholine receptor using photoactivatable derivatives of neurotoxin II from Naja naja oxiana. Biochemistry. 1992;31: 8239–8244. 1525162 10.1021/bi00150a017

[41] Utkin YN, Kobayashi Y, Hucho F, Tsetlin VI. Relationship between the binding sites for an alpha-conotoxin and snake venom neurotoxins in the nicotinic acetylcholine receptor from Torpedo californica. Toxicon. 1994;32: 1153–1157. 7801351 10.1016/0041-0101(94)90399-9

[42] Gaydukov AE, Bogacheva PO, Tarasova EO, Balezina OP. The mechanism of choline-mediated inhibition of acetylcholine release in mouse motor synapses. Acta Naturae. 2014;6: 110–115. PMC4273098 25558401

[43] Lesovoy DM, Bocharov EV, Lyukmanova EN, Kosinsky YA, Shulepko MA, Dolgikh DA, et al Specific membrane binding of neurotoxin II can facilitate its delivery to acetylcholine receptor. Biophys J. 2009;97: 2089–2097. doi: 10.1016/j.bpj.2009.07.037 19804741 10.1016/j.bpj.2009.07.037 PMC2756353

[44] Shenkarev ZO, Lyukmanova EN, Paramonov AS, Panteleev PV, Balandin SV, Shulepko MA, et al Lipid-protein nanodiscs offer new perspectives for structural and functional studies of water-soluble membrane-active peptides. Acta Naturae. 2014;6: 84–94. 25093115 PMC4115230

[45] Sundlass NK, Eller CH, Cui Q, Raines RT. Contribution of electrostatics to the binding of pancreatic-type ribonucleases to membranes. Biochemistry. 2013;52: 6304–6312. doi: 10.1021/bi400619m 23947917 10.1021/bi400619m PMC3839965

[46] Subramanian M, Jutila A, Kinnunen PK. Binding and dissociation of cytochrome c to and from membranes containing acidic phospholipids. Biochemistry. 1998;37: 1394–1402. doi: 10.1021/bi9716581 9477968 10.1021/bi9716581

